# Acute Respiratory Distress Syndrome After Severe Acute Respiratory Syndrome Coronavirus (SARS-CoV-2) Vaccination: Findings From an Autopsy

**DOI:** 10.7759/cureus.70370

**Published:** 2024-09-28

**Authors:** Fumiya Kawano, Mikiya Kato, Yasuaki Tsuchida, Jumpei Okawa, Hideki Arimoto

**Affiliations:** 1 Cardiology, Nippon Medical School Hospital, Tokyo, JPN; 2 Emergency Medicine, Iseikai International General Hospital, Osaka, JPN; 3 Pathology, Iseikai International General Hospital, Osaka, JPN

**Keywords:** acute respiratory distress syndrome [ards], autopsy, corona virus, covid-19, sars cov-2, vaccination

## Abstract

A male patient in his 80s was brought to our emergency room with a one-day history of general malaise and cough. He had received a severe acute respiratory syndrome coronavirus-2 (SARS-CoV-2) vaccination seven days earlier. He initially presented with respiratory failure, and an alveolar hemorrhage was suspected. The patient was intubated and admitted to the intensive care unit. We suspected that the alveolar hemorrhage was caused by acute respiratory distress syndrome (ARDS). Treatment including high-dose corticosteroid therapy and venovenous extracorporeal membrane oxygenation (ECMO) was initiated, however, the patient did not respond to treatment and died 35 days after admission. Pathological autopsy revealed diffuse alveolar damage (DAD) and idiopathic diffuse alveolar hemorrhage (DAH) consistent with ARDS. ARDS should be considered as a differential diagnosis when a patient develops respiratory symptoms after receiving the SARS-CoV-2 vaccine.

## Introduction

In recent years, vaccines have been developed against coronavirus disease 2019 (COVID-19), and more than 10 billion doses have been administered worldwide. However, the cumulative toxicity of serial injections of COVID-19 was not studied as part of the regulatory approval process [1]. The adverse events associated with SARS-CoV-2 vaccines have recently drawn attention. Myocarditis, thrombocytopenia, and thromboembolism are rare but well-known side effects, whereas vaccine-induced acute respiratory distress syndrome (ARDS) is not commonly seen [2,3]. Based on our literature search, there are several reports presenting cases of ARDS developing after SARS-CoV-2 vaccination; however, most patients were thought to have developed ARDS due to coexisting infectious diseases [4,5]. Only two reports have concluded that ARDS was induced by SARS-CoV-2 vaccination [6,7].

## Case presentation

A man in his 80s was transported to the emergency room of our hospital with a one-day history of worsening general malaise and cough. His medical history included only hypertension. He developed symptoms seven days after receiving his fifth dose of the SARS-CoV-2 vaccine (BNT162b2-mRNA); he had not experienced similar symptoms from the first to the fourth vaccine doses. On arrival at our hospital, the patient was in a state of respiratory failure. Initial vital signs were as follows: SpO2 of 99% (oxygen mask with reservoir: 15 L/min), respiratory rate of 28 breaths/min, blood pressure of 142/76 mmHg, and heart rate of 109 bpm. Coarse crackles were heard in both lungs, and bloody sputum was observed by airway suction. He had no physical findings suggestive of connective tissue disease such as skin rash or arthritis. The patient was intubated in the emergency room, and mechanical ventilation was initiated. On admission, his P/F ratio was 118 with positive end-expiratory pressure (PEEP) of 10 cmH2O.

**Figure 1:**
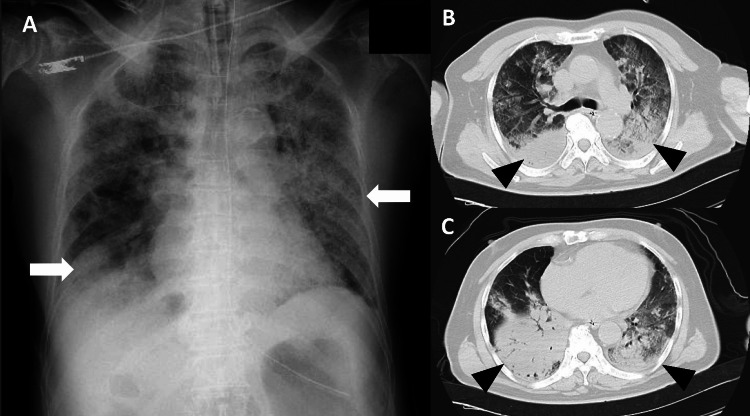
Images of chest x-ray and computed tomography (CT) Chest radiograph showing extensive consolidation and ground-glass opacity in both lungs (A, white arrows), and the axial view of the chest CT scan showing infiltrates in the peripheral areas of the bilateral lower lobes in addition to similar findings on radiography (B, C, black arrowheads).

SARS-CoV-2 polymerase chain reaction (PCR) and rapid antigen testing of nasopharyngeal samples were negative. Laboratory tests revealed lactate dehydrogenase at 379 U/L, C-reactive protein at 19.38 mg/dL, white blood cell count of 12,010 cells/μL, neutrophils at 10,100/μL; 84.1%, lymphocytes at 1057/μL; 8.8%, D-dimer at 6.4 μg/mL, brain natriuretic peptide at 121.8 pg/mL, and Krebs von den Lungen-6 at 520 U/mL (Table 1). Chest radiography and computed tomography (CT) showed dense consolidation and diffuse ground-glass opacity in the dependent regions of the bilateral lungs along with infiltrates in the peripheral areas of the bilateral lower lobes (Figure 1). Echocardiography performed in the emergency room revealed no abnormalities in cardiac function.

**Table 1:** Initial laboratory data on the patient PT-INR: prothrombin time-international normalized ratio, APTT: activated partial thromboplastin time, TP: total protein, Alb: Albumin, AST: aspartate aminotransferase, ALT: alanine aminotransferase, LDH: lactate dehydrogenase, BUN: blood urea nitrogen, Cr: creatinine, CRP: c-reactive protein, BNP: brain natriuretic peptide, KL-6: Krebs von den lungen-6.

Test	Observed Value	Reference Range
White Blood Cell	12,010 /μL	4,000-9,000 /μL
Neutrophil	10,100 /μL; 84.1%	37-73 %
Lymphocyte	1057 /μL; 8.8%	25-45 %
Hemoglobin	12.4 g/dL	13.5-17.0 g/dL
Platelet	228,000 /μL	150,000-400,000 /μL
PT-INR	1.08	0.9-1.1
APTT	25 seconds	24-34 seconds
D-dimer	6.4 μg/mL	0-1 μg/mL
TP	7 g/dL	6.5-8.2 g/dL
Alb	3.6 g/dL	3.7-5.5 g/dL
AST	37 U/L	8-38 U/L
ALT	31 U/L	4-44 U/L
LDH	379 U/L	124-222 U/L
BUN	27.5 mg/dL	8-20 mg/dL
Cr	1.31 mg/dL	0.36-1.06 mg/dL
CRP	19.38 mg/dL	0-0.3 mg/dL
BNP	121.8 pg/mL	0-18.4 pg/mL
KL-6	520 U/mL	0-499 U/mL

Initially, we considered the possibility of acute interstitial pneumonia (AIP) or bacterial or viral pneumonia. However, microbiological tests, including sputum and blood cultures, were negative. All tests available at our hospital for infectious diseases, such as Influenza A/B, *Mycoplasma pneumoniae*, *Legionella pneumophila*, and HIV, returned negative results. Immunological tests also showed that all autoantibodies, including anti-neutrophil cytoplasmic antibody and anti-glomerular basement membrane antibody antibodies, were negative. Based on these findings, we excluded AIP and other forms of pneumonia. On the other hand, the patient met the Berlin criteria for ARDS [8]. Because of this, we diagnosed the patient with ARDS and assumed that this respiratory condition was the cause of the alveolar hemorrhage.

**Figure 2:**
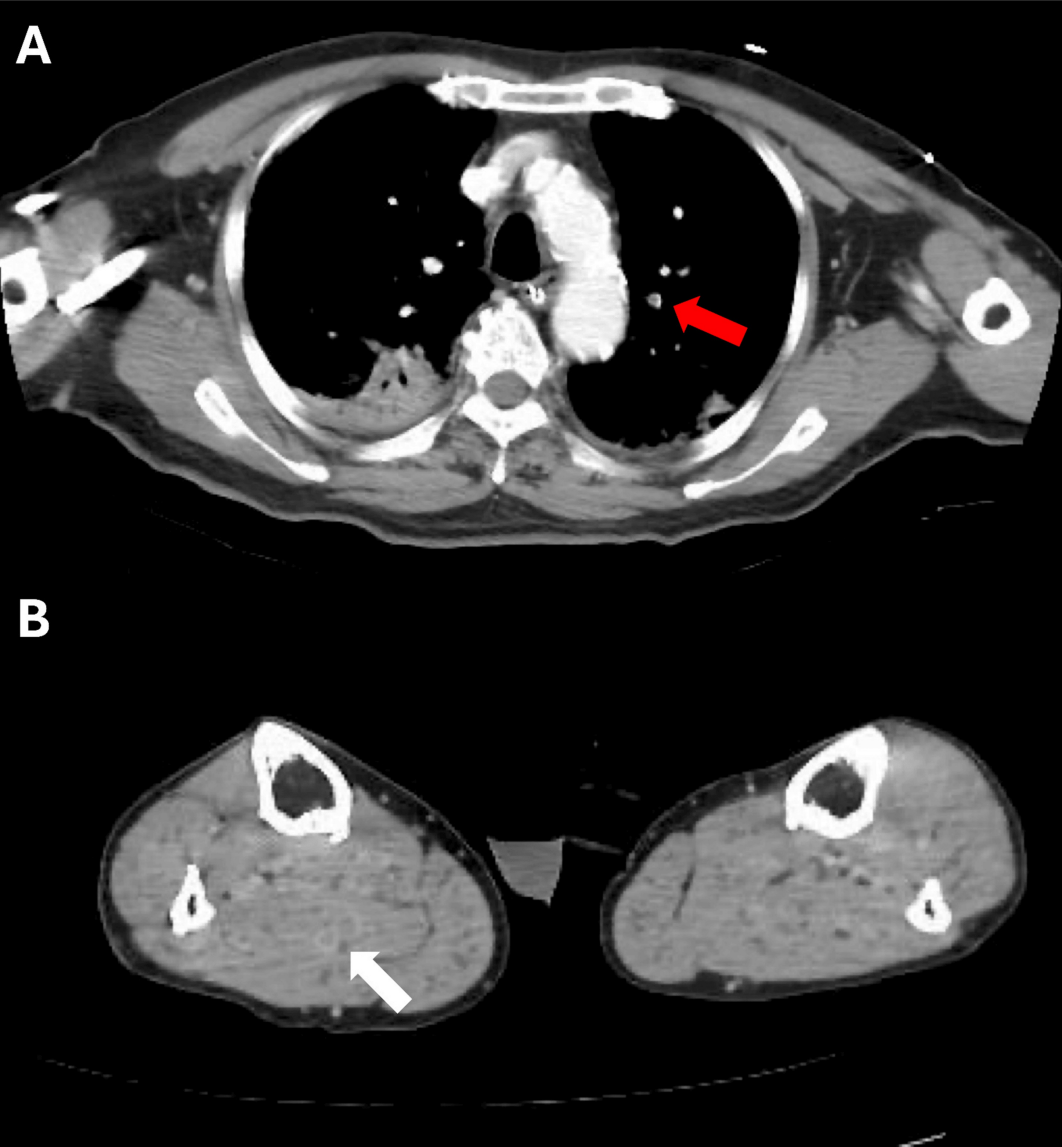
Contrast-enhanced CT on hospital day 10 The axial view of the contrast-enhanced CT scan on hospital day 10 revealed a small pulmonary thromboembolism (A, red arrow) and deep venous thrombosis in his right lower leg (B, white arrow).

Because we initially could not rule out the possibility of AIP, we initiated two cycles of high-dose corticosteroid therapy (first cycle: methylprednisolone 500 mg from hospital days 1 to 3, followed by prednisolone 50 mg from days 4 to 11; second cycle: methylprednisolone 1000 mg from days 12 to 14, followed by prednisolone 50 mg from days 15 to 35). As bacterial pneumonia could not initially be ruled out, a combination of tazobactam/piperacillin (4.5g/day) and levofloxacin (500 mg/day) was also initiated on day one. On hospital day 10, contrast-enhanced CT also revealed a small pulmonary thromboembolism (Figure 2A, red arrow) and deep venous thrombosis in his right lower leg (Figure 2B, white arrow), and anticoagulant therapy with heparin was initiated.

The patient did not respond to treatment. On hospital day 10, tracheostomy was performed. Venovenous extracorporeal membrane oxygenation (ECMO) was initiated on day 12 Despite extensive and prolonged management in the ICU, the patient’s respiratory condition did not improve. On day 35, we determined that the patient would likely not improve, and ECMO was discontinued with agreement from his family; the patient died the same day. A pathological autopsy was performed at the family’s request.

**Figure 3:**
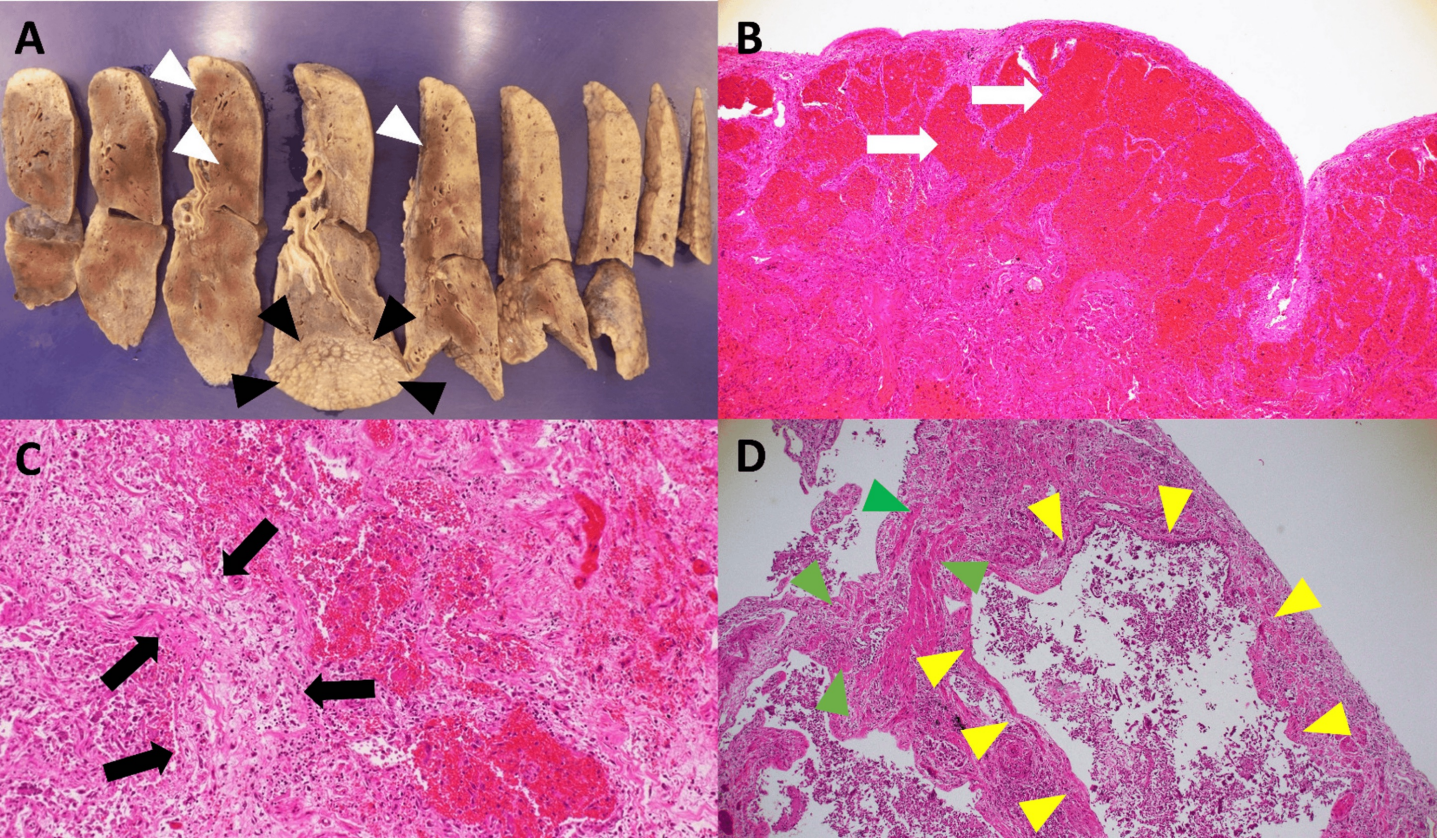
Pathological findings by autopsy Gross appearance of the cut lung surfaces (left lobe) revealed a honeycomb appearance (black arrowheads) and spot hemorrhage (white arrowheads), and overall poor air content was found (macroscopic findings) (A). There was diffuse alveolar hemorrhage (white arrows) in parts of the lungs (Hematoxylin and Eosin (H&E) staining, original magnification ×40) (B) and extensive interstitial fibrosis with uniform phase and distribution (black arrows) (H&E staining, original magnification ×100) (C). There was also extensive traction bronchiectasis (yellow arrowheads) in the peripheral lung fields, and honeycomb formations (green arrowheads) in parts of both lower lobes of the lungs (H&E staining, original magnification ×40) (D).

Gross examination of the cut surface of the lungs showed a honeycomb appearance, spot hemorrhage, and overall poor air content (Figure 3A). Microscopically, the specimen showed diffuse alveolar hemorrhage (DAH) in parts of the lungs (Figure 3B) and extensive interstitial fibrosis with a uniform phase and distribution (Figure 3C). There was also extensive traction bronchiectasis in the peripheral lung fields as well as honeycomb formations in parts of the bilateral lower lobes (Figure 3D). A microscopic examination revealed no evidence of connective tissue disease or vasculitis. SARS-CoV-2 PCR testing of the lung tissue was negative. No cytomegaloviral, bacterial, or fungal infections were detected in the autopsy tissues. Based on the above findings, the main pathological diagnoses were diffuse alveolar damage (DAD) at the organic stage, and idiopathic DAH, which were consistent with the late-stage pathological changes of ARDS.

## Discussion

ARDS is a clinical syndrome that can result from a variety of etiologies such as sepsis, pancreatitis, aspiration, shock, and trauma [9]. Based on our literature search, several case studies have shown ARDS development after vaccination (such as influenza vaccine); however, most studies have shown that ARDS is induced by infection [4]. ARDS associated with vaccination (SARS-CoV-2 or other vaccines) is rare; only two studies have concluded that the development of ARDS is associated with SARS-CoV-2 vaccination. The first case involved a woman in her 80s who developed dyspnea several hours after receiving the second dose of the mRNA SARS-CoV-2 vaccine. She was diagnosed with ARDS and treated with high-dose corticosteroids; she died on hospital day 18, and the autopsy revealed very early phase diffuse alveolar damage throughout the lungs. No other lung pathology was observed [6]. The second patient was a pregnant woman in her 20s. The day after the second dose of the mRNA SARS-CoV-2 vaccination, the patient developed fever, bilateral pleuritic chest pain, exertional dyspnea, diaphoresis, and palpitations. She was also diagnosed with ARDS and recovered after a two-week course of high-dose corticosteroids [7].

The mechanism underlying the occurrence of ARDS after SARS-CoV-2 vaccination remains unclear. ARDS is generally caused by a complex interplay between the immune and inflammatory systems. The inflammatory or exudative phase begins shortly after an inciting insult activates and amplifies the response of the innate immune system [10]. Vaccination may trigger this activation. The innate immune system in individuals with a specific immunogenetic predisposition reacts abnormally to vaccine molecules, for example, mRNA [11]. Another possible mechanism for autoimmune disease development following vaccination is molecular mimicry, in which the antibody against the spike protein of SARS-CoV-2 cross-reacts with human tissue antigens [12]. Autoantibodies are being evaluated as potential mechanisms for various adverse events following COVID-19 vaccination such as thrombocytopenia [13].

Interstitial lung disease can be considered as a differential diagnosis for ARDS. Many cases of acute exacerbation of chronic interstitial lung disease after SARS-CoV-2 vaccination have been reported [14]. We doubt that this was the case in our patient because he had no history of lung disease and developed respiratory symptoms along with alveolar hemorrhage; additionally, we excluded other diseases that could cause alveolar hemorrhage using various tests. Pathological results also did not show findings suggestive of chronic lung diseases such as idiopathic pulmonary fibrosis.

There are some limitations. First, we could not attempt to detect vaccine mRNA in lung tissue and to identify Spike protein in the tissue. There are a report recommending testing for the presence of the Spike protein and vaccine-derived mRNA in tissue samples to better understand the involvement of COVID-19 vaccines in adverse events [15]. Such testing could have allowed for a more detailed investigation. Second, we were unable to find out the batch of vaccines he received. If the batch of vaccine was known, it may have been possible to search for reports of adverse events caused by the same batch of vaccine, which could have been examined in more detail.

## Conclusions

This case reports a patient who developed ARDS after receiving the SARS-CoV-2 vaccine, with autopsy findings confirming the diagnosis. Despite intensive care, including corticosteroids and ECMO, the patient did not survive, highlighting the severity of vaccine-induced ARDS. The exact mechanism remains unclear, possibly involving abnormal immune responses, and further research is needed to understand the link between vaccination and ARDS. Ongoing monitoring is recommended for individuals receiving multiple mRNA vaccine doses, as cumulative toxicity may contribute to such severe outcomes.
